# Selective vulnerability of hippocampal sub-regions in patients with subcortical vascular mild cognitive impairment

**DOI:** 10.1007/s11682-024-00881-y

**Published:** 2024-04-20

**Authors:** Jianxiang Chen, Jianjun Wang, Ke Duan, Xinbei Li, Zhongxian Pan, Jinhuan Zhang, Xiude Qin, Yuanming Hu, Hanqing Lyu

**Affiliations:** 1 Department of Radiology, The Fourth Clinical Medical College, Shenzhen Traditional Chinese Medicine Hospital, Guangzhou University of Chinese Medicine, Shenzhen, China; 2 Department of Neurology and Psychology, The Fourth Clinical Medical College, Shenzhen Traditional Chinese Medicine Hospital, Guangzhou University of Chinese Medicine, Shenzhen, China; 3 Department of Acupuncture and Moxibustion, The Fourth Clinical Medical College, Shenzhen Traditional Chinese Medicine Hospital, Guangzhou University of Chinese Medicine, Shenzhen, China

**Keywords:** Hippocampus, Segmentation, Vascular mild cognitive impairment, Subcortical brain regions, Small vessel disease

## Abstract

Early diagnosis of subcortical vascular mild cognitive impairment (svMCI) is clinically essential because it is the most reversible subtype of all cognitive impairments. Since structural alterations of hippocampal sub-regions have been well studied in neurodegenerative diseases with pathophysiological cognitive impairments, we were eager to determine whether there is a selective vulnerability of hippocampal sub-fields in patients with svMCI. Our study included 34 svMCI patients and 34 normal controls (NCs), with analysis of T1 images and Montreal Cognitive Assessment (MoCA) scores. Gray matter volume (GMV) of hippocampal sub-regions was quantified and compared between the groups, adjusting for age, sex, and education. Additionally, we explored correlations between altered GMV in hippocampal sub-fields and MoCA scores in svMCI patients. Patients with svMCI exhibited selectively reduced GMV in several left hippocampal sub-regions, such as the hippocampal tail, hippocampal fissure, CA1 head, ML-HP head, CA4 head, and CA3 head, as well as decreased GMV in the right hippocampal tail. Specifically, GMV in the left CA3 head was inversely correlated with MoCA scores in svMCI patients. Our findings indicate that the atrophy pattern of patients with svMCI was predominantly located in the left hippocampal sub-regions. The left CA3 might be a crucial area underlying the distinct pathophysiological mechanisms of cognitive impairments with subcortical vascular origins.

## Introduction

Cognitive impairment is one of the primary public health challenges in our era (Hampel & Lista, 2016). Vascular-related cognitive decline is the second most prevalent cause, and in East Asia, it may take precedence as the primary cause (Iadecola et al., 2019). Subcortical vascular mild cognitive impairment (svMCI) is a type of cognitive impairment caused by cerebral small vessel disorder (Zotin et al., 2021). svMCI is characterized by a gradual onset and is mainly associated with attention and executive function impairment (Hamilton et al., 2021; Zotin et al., 2021). As the prodromal stage of subcortical vascular dementia, svMCI is the most responsive subtype to treatment through modification of vascular risk factors (Iadecola et al., 2019; Van Der Flier et al., 2018), signifying the early phase of the disease. As a result, identifying svMCI at an early stage is of great importance in clinical practice. The mechanisms underlying cognitive impairment associated with subcortical vascular lesions need to be largely explored.

Hippocampal atrophy is a common structural and pathophysiological change in various cognitive dysfunctions, such as Alzheimer’s disease (AD) (Devanand et al., 2007), post stroke dementia, and subjective cognitive decline (Das et al., 2019; Devanand et al., 2007; Huang et al., 2022). Moreover, dynamic hippocampal atrophy has been observed during healthy human aging (Oschwald et al., 2019). Neurofibrillary tangle deposition and neuronal death are linked to hippocampal shrinkage, which is a significant cause of cognitive impairment in vascular illness (Yamamoto et al., 2021) and is linked to hippocampal atrophy. Even minor vascular disorders can result in hippocampus atrophy and cognitive deterioration (Han et al., 2020). However, the hippocampus was considered a single, homogeneous structure in most previous studies, and potentially useful information about its sub-regions has been discarded. Several distinct sub-regions with various structural and functional profiles have been identified in the bilateral hippocampus (Fraser et al., 2015). Among these, a statistical atlas of the hippocampal using ultra-high resolution ex vivo magnetic resonance imaging (MRI) combined with in vivo data in the FreeSurfer 6 and above version (https://surfer.nmr.mgh.harvard.edu/fswiki/rel7downloads) has been widely adopted to investigate structural alterations of the hippocampal at the sub-regional level in many brain disorders (Bai et al., 2019; Christidi et al., 2019; Xu et al., 2022). Previous studies have demonstrated that selective atrophy of hippocampal sub-regions is associated with particular brain diseases. For example, the cornu ammonis 2/3 subfield and the hippocampus-amygdala transition area are the most affected regions in amyotrophic lateral sclerosis in contrast to AD, where the presubiculum and subiculum are the most vulnerable regions (Christidi et al., 2019). Moreover, some studies reported that patients with mild cognitive impairment showed smaller gray matter volumes, primarily in cornu ammonis CA1-2 volumes, compared to healthy controls (Mueller et al., 2010). Considering the various etiologies of svMCI, whether there is a selective vulnerability of hippocampal sub-fields is extremely worth to be further explored.

Consequently, we hypothesized that patients with svMCI would exhibit distinctive atrophy patterns of volumetric changes in hippocampal sub-regions. To test this hypothesis, we obtained T1 images from 34 svMCI patients and 34 normal controls, and performed hippocampal segmentation of these T1 images using FreeSurfer 7.1. The gray matter volume (GMV) of the hippocampal sub-regions was measured and compared between groups after adjusting for age, sex and educational level. Finally, Pearson’s correlation was performed between changed GMV and MoCA in the svMCI.

## Methods and materials

### Participants

This research was conducted in the Shenzhen Traditional Chinese Medicine Hospital and authorized by their Institutional Review Board (K2021-041). The diagnosis of MCI was made by two neurological physicians with more than five years of experience (XDQ and JJW) based on Peterson’s criteria as follows (Jia et al., 2016; Petersen et al., 1999). The inclusion criteria required cognitive complains in memory and/or other cognitive domains for at least three months, objective cognitive impairments not meeting the DSM-IV criteria for dementia, an abnormal clinical dementia rating (CDR) of ≥ 0.5 on at least one domain and a global score ≤ 0.5 (Saygin et al., 2017), a Montreal cognitive assessment (MoCA) score of < 26 (Nasreddine et al., 2005; Sun et al., 2023), and normal or near-normal performance of daily life activities. The following cases were excluded from the study: individuals with a history of psychiatric disorder in any two lines of first- to third-degree biological family; those with a history of strokes or transient ischemic attack within three months; those with a history of seizures, schizophrenia, or major depressive disorder; those with an inherited or inflammatory small vessel illness; those who previously suffered from a head injury with unconsciousness; those who used nootropics, such as Donepezil and Kabalatine; those with significant clinical or surgical conditions; those with physical disabilities, including aphasia, blindness, or hemiplegia, which can prevent the fulfillment of neuropsychological tests; and those with contraindications for MRI.

Patients with svMCI needed to meet at least one of the following criteria: three or more supratentorial subcortical minor infarcts with or without white matter lesions of any severity; moderate to severe white matter lesion (score ≥ 2 on the Fazekas rating scale) with or without small infarcts; or one or more subcortical minor infarcts strategically positioned in the caudate nucleus, globus pallidus, or thalamus. The evaluation of MRI images was done by two experienced radiologists (JXC and HQL), who were unaware of the participants’ identities. In cases of disagreement, they would re-evaluate the images after discussion.

The NCs were recruited through advertisements from the community of Shenzhen and had no prior history of neurological or psychiatric disorders, no cognitive issues, and their conventional brain MRI images were normal. Herein, 34 svMCI and 34 NCs were involved. The demographics and clinical characteristics of all participants are presented in Table 1.

**Table 1:** Demographic data and clinical measures

Groups	svMCI	NC	T value	*p* value
Subjects	34	34	-	*-*
Age (mean ± SD)	63.32 ± 6.81	61.4 ± 4.96	1.505	0.137^a^
Sex (male/female)	16/18	14/20	-	0.807^b^
Education (mean ± SD)	8.29 ± 3.77	9.94 ± 3.66	-1.826	0.072^a^
MoCA (mean ± SD)	19.50 ± 2.14	27.91 ± 1.04^c^	-17.40	< 0.001^a^
eTIV	1452767.25 ± 113371.94	1421756.43 ± 128990.46	1.053	0.296 ^a^

Notes: ^a^ represents two sample t tests; ^b^ represents χ2; and ^c^ only data of 24 people are available. Abbreviations: svMCI, subcortical vascular mild cognitive impairment; NC, normal controls; SD, standard deviation; MoCA, Montreal cognitive assessment; eTIV, estimated total intracranial volume

### MRI acquisition

MRI scanning was performed on a 3T scanner (GE medical system, MR750) with a 32-channel head coil. The scanning criteria of T1 images were as follows: repetition time = 8.656 ms, echo time = 3.22 ms, inversion time = 450 ms, flip angle = 12, matrix size = 256 × 256, slice thickness = 1 mm, voxel size = 1 × 1 × 1 mm^3^, and sections = 152.

### Image processing

Utilizing the usual “recon-all” process in FreeSurfer 7.1 (http://surfer.nmr.mgh.harvard.edu/), the cortical surface was reconstructed. Overall, the pictures were preprocessed by movement correction, brain retrieval, Talairach transformation, intensity correction, and segmentation of gray matter, white matter, and cerebrospinal fluid from the brain tissue. Additionally, the barrier between gray matter and white matter was tessellated. The segmentation of subcortical structures was further explored using a nonlinear warping atlas.

### Hippocampal segmentation

The hippocampal segmentation was performed using the automated hippocampal module in FreeSurfer 7.1. Specifically, it was carried out using a Bayesian inference technique that employed a probabilistic atlas of the hippocampal formation that was trained on a hybrid data set involving in-vivo ultra-high-resolution (0.1 mm) MRI and ex-vivo autopsied brain MRI from multiple subjects. This probabilistic atlas was registered to T1 images in the individual space for each subject. The published works of Saygin et al. (Saygin et al., 2017), Iglesias et al. (Iglesias et al., 2015), and Van Leemput et al. (Puonti et al., 2016) provided extra details on the segmentation technique. To evaluate the quality of these segmentations, all segmented volumes of participants were visually inspected by a trained operator (JXC). Moreover, hippocampal subfield volumes were plotted and outliers were flagged for a secondary inspection.

### Gray matter volume alterations of hippocampal sub-regions

Two-sample t-tests were employed to identify volume shifts in hippocampal sub-regions adjusting for age, sex, education, and estimated total intracranial volume (eTIV) (Jack et al., 2015; Perrotin et al., 2015) utilizing SPSS Statistics (Armonk, NY: IBM Corp., v.19.0). *p* < 0.05 was judged statistically significant, not corrected.

### Correlation analyses

Pearson’s partial correlation analyses between the volume and MoCA scores of all hippocampal sub-regions that exhibited gray matter atrophy were performed with age, sex, eTIV, and educational level as covariates. The *p* < 0.05 was judged statistically significant, not corrected. These analyses were performed using SPSS 25.

## Results

### Demographics and clinical data

Demographics and clinical data (Table 1) were compared between the two groups using a two-sample t-test. Sex was analyzed using χ^2^. Consequently, we found no significant variation in age (*p* = 0.137), sex (*p* = 0.807), education (*p* = 0.072), or eTIV (*p* = 0.296), but there was a significant difference in MoCA between svMCI and NCs (*p* < 0.001).

### Hippocampal segmentation

**Fig. 1:**
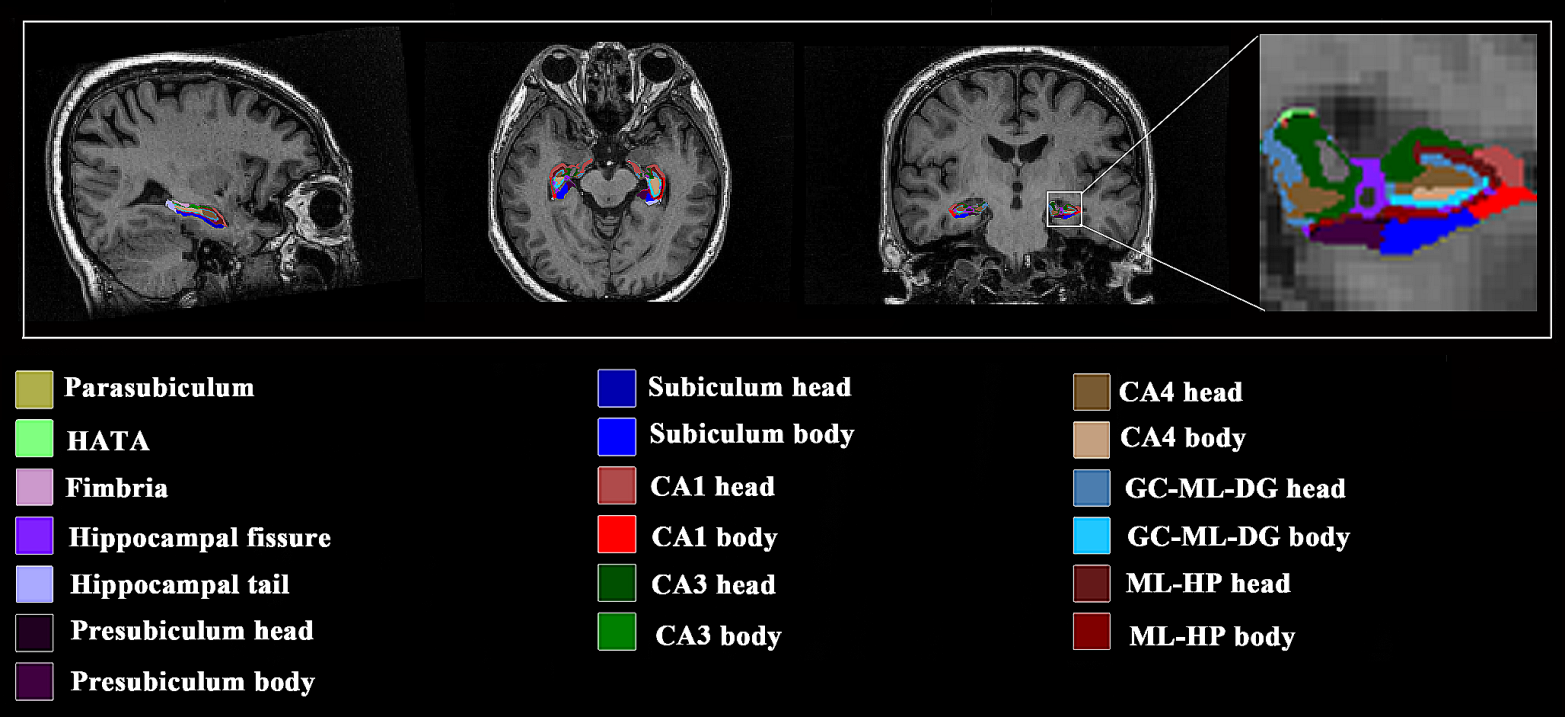
Segmentation results of hippocampus on T1 MRI scans. The images were shown with FSLeyes (https://fsl.fmrib.ox.ac.uk/fsl/fslwiki/FSLeyes). Abbreviations: HATA, hippocampus-amygdala-transition-area; CA, cornu ammonis; GC-ML-DG, granule cell molecular layer of the dentate gyrus; ML-HP, molecular layer hippocampus

Hippocampal segmentation resulted in 19 sub-regions for each hemisphere (Fig. 1): the hippocampal tail, subiculum body, subiculum head, cornu ammonis (CA) 1 body, CA1 head, CA3 body, CA3 head, CA4 head, CA4 body, hippocampal fissure, presubiculum head, presubiculum body, parasubiculum, molecular layer hippocampal (ML-HP) head, ML-HP body, granule cell molecular layer of dentate gyrus (GC-ML-DG) head, GC-ML-DG body, fimbria, and hippocampus-amygdala-transition-area (HATA).

### GMV shifts of hippocampal sub-regions

**Fig. 2:**
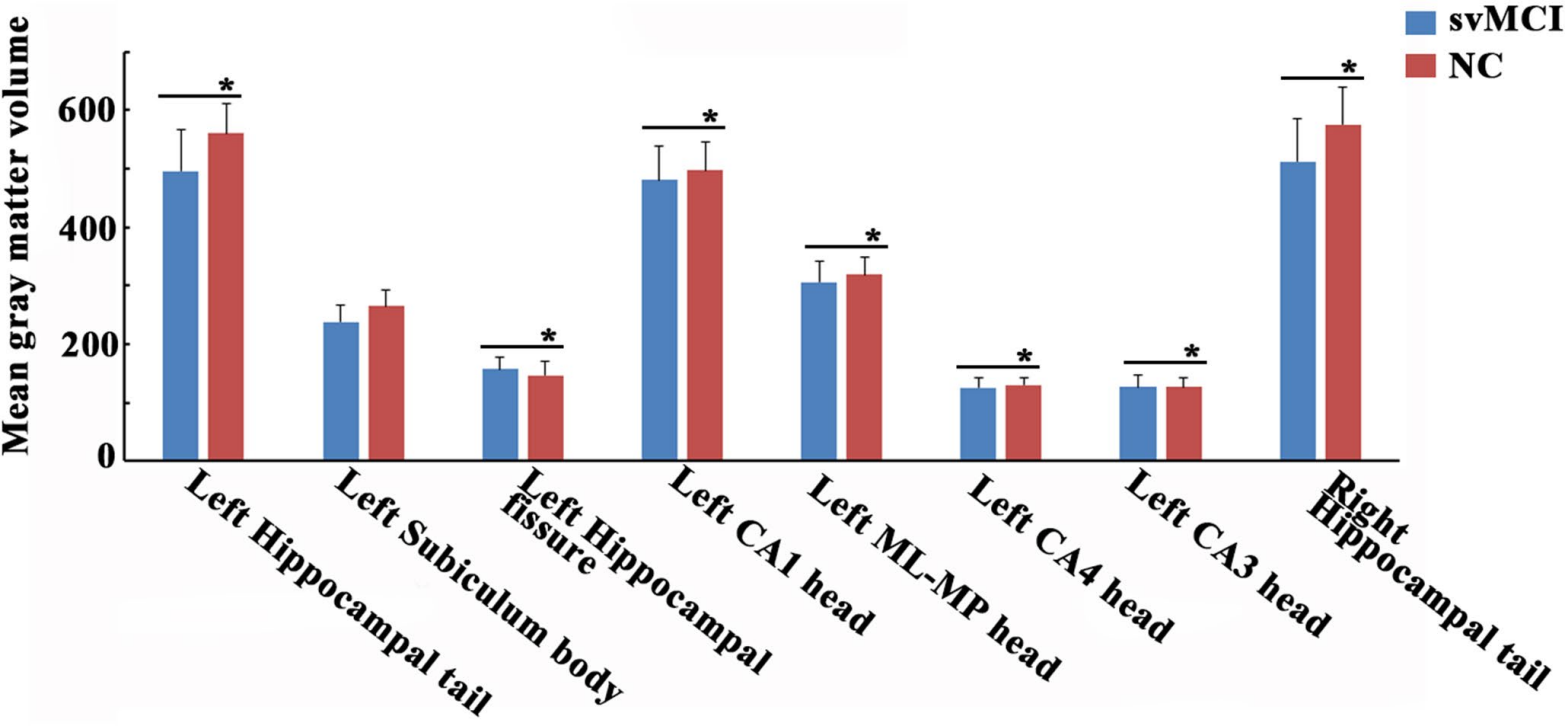
The gray matter volume changes of hippocampal sub-regions. Two-sample t-tests were employed to identify volume changes in hippocampal sub-regions adjusting for age, sex, education, and estimated total intracranial volume (eTIV) between patients with svMCI and NC.* represents significant results with *p*_*uncorrected*_< 0.05

In contrast to NCs, patients with svMCI demonstrated significantly reduced GMV in several left hippocampal sub-regions, including the left hippocampal tail(*p*_*uncorrected*_ = 0.004), left hippocampal fissure (*p*_*uncorrected*_ = 0.040), left CA1 head(*p*_*uncorrected*_ = 0.011), left ML-HP head(*p*_*uncorrected*_ = 0.011), left CA4 head(*p*_*uncorrected*_ = 0.033), and left CA3 head(*p*_*uncorrected*_ = 0.011), but only in the hippocampal tail in the right hippocampal sub-regions(*p*_*uncorrected*_ = 0.016) (Tables 2**and** Fig. 2).

**Table 2:** Gray matter volume alterations in hippocampal sub-regions in the svMCI compared to NC

	Left hemisphere					Right hemisphere				
	svMCI		NC		*P*_uncorrected_	svMCI		NC		*P*_uncorrected_
	Mean	SD	Mean	SD		Mean	SD	Mean	SD	
Hippocampal_tail	495.377	72.177	559.863	51.558	**0.004**	511.749	72.958	575.421	62.772	**0.016**
subiculum_body	238.234	28.503	264.557	28.030	0.074	240.095	29.916	258.934	28.591	0.798
CA1_body	120.012	19.268	123.767	13.955	0.520	131.974	22.049	140.335	19.539	0.833
subiculum_head	168.201	22.844	177.383	26.845	0.287	174.549	31.016	190.663	23.652	0.721
hippocampal_fissure	156.463	21.592	146.402	24.765	**0.040**	169.038	29.652	162.233	33.084	0.074
presubiculum_head	124.865	16.903	132.214	15.031	0.609	126.530	19.880	135.387	13.714	0.436
CA1_head	480.680	57.561	497.243	48.294	**0.011**	507.732	71.656	538.818	55.444	0.139
Presubiculum_body	142.135	25.620	161.748	19.266	0.075	131.796	22.835	147.311	17.979	0.372
Parasubiculum	60.319	14.153	66.112	10.082	0.474	59.715	13.293	62.133	8.678	0.632
Molecular_layer_HP_head	305.395	35.599	319.164	30.507	**0.011**	320.595	46.282	344.297	32.569	0.383
Molecular_layer_HP_body	211.425	24.436	228.456	19.366	0.463	221.300	30.526	244.001	25.795	0.227
GC_ML_DG_head	147.586	20.659	154.438	16.320	0.069	158.758	25.744	167.317	17.359	0.263
CA3_body	92.512	18.978	91.421	14.836	0.100	102.181	16.501	110.013	15.048	0.633
GC_ML_DG_body	132.584	15.783	143.513	12.242	0.278	139.484	18.456	150.738	14.946	0.905
CA4_head	125.817	15.416	129.824	13.563	**0.033**	134.591	21.069	140.135	14.016	0.184
CA4_body	120.706	15.225	127.930	10.482	0.754	127.306	17.455	137.497	13.625	0.633
Fimbria	80.080	25.820	93.447	21.525	0.452	83.359	22.482	89.604	18.467	0.691
CA3_head	126.198	19.975	126.326	16.630	**0.011**	135.813	25.588	136.979	18.126	0.069
HATA	55.193	11.837	57.695	7.179	0.220	57.409	11.060	58.159	6.821	0.094

Abbreviations: HATA, hippocampus-amygdala-transition-area; CA, cornu ammonis; GC-ML-DG, granule cell molecular layer of dentate gyrus; ML-HP, molecular layer hippocampus

### Results of correlation analyses

**Fig. 3:**
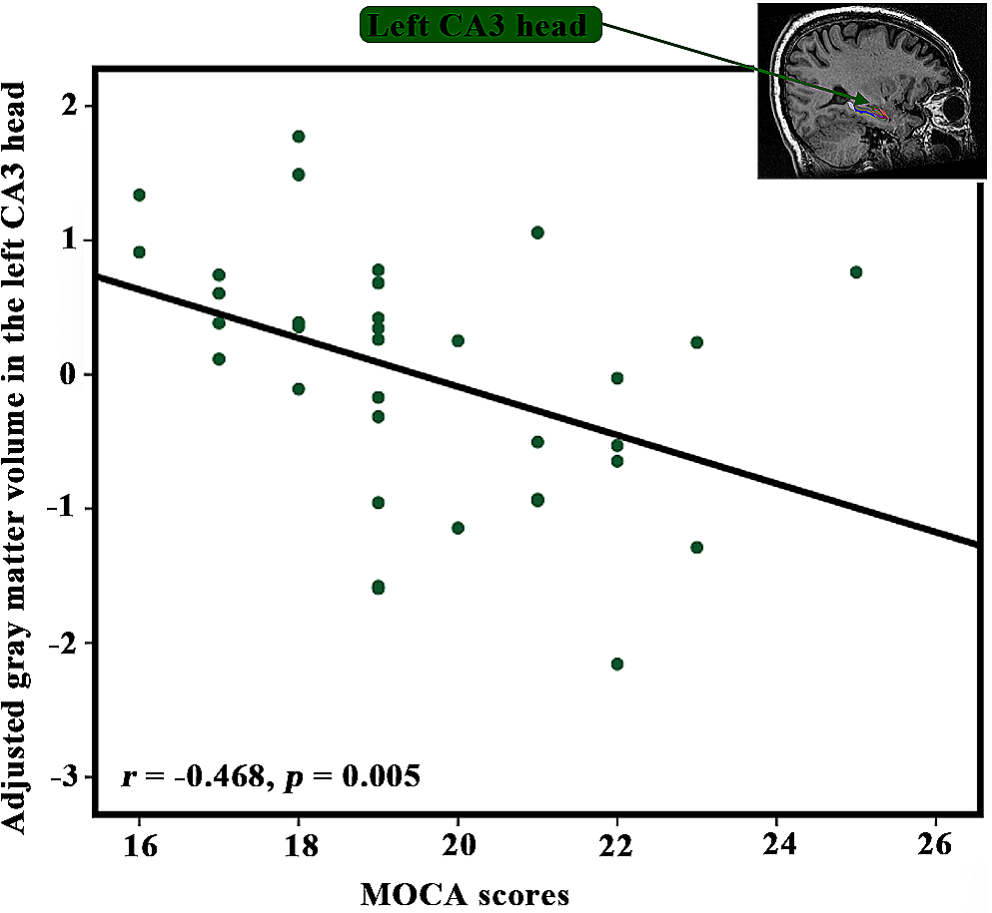
Relationship between gray matter volume and cognitive impairments in patients with svMCI. The gray matter volume of left CA3 head was adjusted by regressing out covariates of age, sex, eTIV, and educational level. Then, Pearson’s correlation analyses between the volume of left CA3 head and MoCA scores in the patients with svMCI were performed. Abbreviations: MoCA, Montreal cognitive assessment

The GMV in the left CA3 head was negatively correlated with MoCA scores in the svMCI (*r* = -0.468, *p* = 0.005, Fig. 3). However, no significant correlations were observed between GMV of other altered hippocampal sub-regions and MoCA scores in the svMCI (Table 3).

**Table 3:** Results of correlation analyses

Subregions	P_uncorrected_	R values
Left Hippocampal_tail	0.782	-0.052
Left Hippocampal_fissure	0.774	0.055
Left CA1_head	0.485	-0.132
Left Molecular_layer_HP_head	0.976	-0.006
Left CA4_head	0.180	-0.251
Left CA3_head	0.005	-0.498
Right Hippocampal_tail	0.201	0.240

## Discussion

Herein, we identified that the svMCI group presented hippocampal atrophy primarily in the right hippocampal tail and several left hippocampal sub-regions, such as the hippocampal tail, hippocampal fissure, CA1 head, ML-HP head, CA4 head, and CA3 head. Moreover, the GMV in the left CA3 head was negatively associated to the MoCA scores in patients with svMCI. Our results confirm a selective vulnerability of hippocampal sub-fields in patients with svMCI, which adds to our understanding of the pathophysiological mechanisms of cognitive impairment with subcortical vascular origins.

Notably, the findings of this study have demonstrated a discernible susceptibility of bilateral hippocampal sub-regions, with a notable emphasis on the left. Consistent with this finding, a previous study reported that hippocampal atrophy in the svMCI group was more lateralized towards the left hemisphere, particularly in the left subiculum and presubiculum (Li et al., 2016). Furthermore, the shape of the left anterior hippocampus was reduced in patients with svMCI, while those with moderate-to-severe clinical severities had a reduction in the shape of both anterior hippocampi (Thong et al., 2014). Previous research with more severe cognitive impairments reported more remarkable morphological alterations in the right hippocampal sub-regions (He et al., 2022). In healthy elderly adults, the right hippocampus tends to exhibit a larger volume than the left hippocampus, with the left hippocampus being more susceptible to vascular pathology than the right (Hou et al., 2013; Sarica et al., 2018; Shi et al., 2009; Woolard & Heckers, 2012). Furthermore, the asymmetry analysis in specific brain regions was observed to be sensitive for predicting cognitive decline (Long et al., 2018). It is possible to speculate that the left hippocampus might be more sensitive than the right one in the disease progression of svMCI.

We identified alterations in CA1, ML, CA4, and CA3 in the individuals with svMCI. These results were similar to previous studies on amnestic mild cognitive impairment (Kang et al., 2018), another sub-type of mild cognitive impairment, which shared similar symptoms especially memory impairments. Hippocampal CA1 pyramidal neurons play a crucial role in the memory circuit of the medial temporal lobe and are particularly susceptible to damage during the course of Alzheimer’s disease. Meanwhile, research has depicted that the CA1 subfield of the hippocampus in gerbils appears to be vulnerable to anoxic-ischemic insults (Huang et al., 2022; Kirino & Sano, 1984). Further, autopsy studies of patients with microvascular pathology have demonstrated notable atrophy and neuronal loss in the CA1 region (Kril et al., 2002). Consequently, alterations identified in neurodegenerative and vascular cognitive impairments may imply a common pathological mechanism for cognitive decline. Meanwhile, the current investigation revealed that the hippocampal tail, hippocampal fissure, and ML-HP head displayed discernible features that could differentiate svMCI from control subjects. This suggests that the subcortical vascular origin of this condition may result in a distinctive pattern of hippocampal damage. However, different whole hippocampal volumes derived from automatically or manually segmented MRI scans have been published (Pluta et al., 2012; Yushkevich et al., 2010). It is important to note that it is unsuitable to directly compare our results to previous ones if different hippocampal subfields were used. In this regard, we used the latest one, which is built with ultra-high resolution ex vivo MRI data (∼ 0.1 mm isotropic) and ex-vivo autopsied brain MRI from multiple subjects.

Unexpected, we identified a negative correlation between decreased GMV in the left CA3 head and MoCA scores in individuals with svMCI. The hippocampus CA3 is primarily responsible for memory and cognitive control and is composed of pyramidal neurons (Kesner, 2013; Nakashiba et al., 2009; Suthana et al., 2015). The arterial vascularization of the hippocampus depends on the collateral branches of the posterior cerebral artery and the anterior choroidal artery, whereas the CA3 region may only be supplied by the large dorsal hippocampal artery (Tatu & Vuillier, 2014). As a consequence, brain ischemia has a particularly detrimental impact on CA3, one of the most vulnerable subregions of the hippocampus (Vatrinet et al., 2017). Hypoxia in the CA3 area of the hippocampus may impede energy production, lead to hippocampal degeneration, and ultimately result in neural malfunction by triggering inflammation and disrupting the integrity of the blood-brain barrier (Liu et al., 2022). Since all subcortical infarcts were very small (< 20 mm) and white matter lesions (WMLs) were around the ventricle or randomly distributed in the whole brain, it is extremely difficult to obtain all lacunas and microvascular lesions and calculate WMH volume for each subject. Thus, we did not explore the relationship between WMH volume and adjusted CA3 volume of the subjects in this study to exclude the possibility that CA3 might be susceptible to ischemic lesions. Additional attention should be paid to explain the negative relationship between CA3 volume and MoCA in the svMCI.

## Limitations

This research has some drawbacks. First, the relatively small sample size with heterogeneous vascular factors by nature could reduce the external validity of current findings. A larger sample size with multi-center is necessary to replicate our results. Second, the cross-sectional research was unable to establish a cause-and-effect connection between neuroimaging alterations and cognitive deficits. Third, this study focused on the structural modifications of hippocampus sub-regions. Further studies considering the relationship between the hippocampus and the rest of the whole brain are required. Fourth, in the analysis of the relationship between changes in imaging and clinical symptoms, specific focus on regions such as executive function and their correlation with imaging changes was unexplored. Additionally, future studies should consider including information on amyloid-beta deposition and APOE genotyping, as these were important biomarkers of Alzheimer’s disease. Finally, the significant *p* value was not adjusted for multiple comparisons. More caution is needed for the explanation of our results.

## Conclusions

In our investigation, we observed several modifications in GMV that were primarily localized in the left hippocampal sub-regions of individuals with svMCI. Notably, a reduction in GMV within the left CA3 head sub-region was significantly associated with disease severity. Our findings reveal distinct patterns of atrophy within the hippocampal subfields that underlie the pathophysiological mechanisms of cognitive impairment in patients with svMCI. These results may serve as a valuable resource for future investigations aimed at elucidating svMCI pathogenesis.

## Data Availability

The datasets generated for this study are available on request to the corresponding author.

## References

[CR1] Bai, T., Wei, Q., Xie, W., Wang, A., Wang, J., Gong-Jun, J., & Tian, Y. (2019). Hippocampal-subregion functional alterations associated with antidepressant effects and cognitive impairments of electroconvulsive therapy. *PSYCHOLOGICAL MEDICINE*, *49*(8), 1357–1364. 30229715 10.1017/S0033291718002684 PMC6518386

[CR2] Christidi, F., Karavasilis, E., Rentzos, M., Velonakis, G., Zouvelou, V., Xirou, S., & Ferentinos, P. (2019). Hippocampal pathology in amyotrophic lateral sclerosis: Selective vulnerability of subfields and their associated projections. *Neurobiology Of Aging*, *84*, 178–188. 31629116 10.1016/j.neurobiolaging.2019.07.019

[CR3] Das, T., Hwang, J. J., & Poston, K. L. (2019). Episodic recognition memory and the hippocampus in Parkinson’s disease: A review. *Cortex*, *113*, 191–209. 30660957 10.1016/j.cortex.2018.11.021 PMC6445686

[CR4] Devanand, D., Pradhaban, G., Liu, X., Khandji, A., De Santi, S., Segal, S., & Mayeux, R. (2007). Hippocampal and entorhinal atrophy in mild cognitive impairment: Prediction of Alzheimer disease. *Neurology*, *68*(11), 828–836. 17353470 10.1212/01.wnl.0000256697.20968.d7

[CR5] Fraser, M. A., Shaw, M. E., & Cherbuin, N. J. N. (2015). A systematic review and meta-analysis of longitudinal hippocampal atrophy in healthy human ageing. 112, 364–374. 10.1016/j.neuroimage.2015.03.035 25800208

[CR6] Hamilton, O. K., Backhouse, E. V., Janssen, E., Jochems, A. C., Maher, C., Ritakari, T. E., & Wardlaw, J. M. (2021). Cognitive impairment in sporadic cerebral small vessel disease: A systematic review and meta-analysis. *Alzheimer’s & Dementia*, *17*(4), 665–685. 10.1002/alz.12221 PMC8593445 33185327

[CR7] Hampel, H., & Lista, S. (2016). The rising global tide of cognitive impairment. *Nature Reviews Neurology*, *12*(3), 131–132. 26782338 10.1038/nrneurol.2015.250

[CR8] Han, J. W., Maillard, P., Harvey, D., Fletcher, E., Martinez, O., Johnson, D. K., & DeCarli, C. (2020). Association of vascular brain injury, neurodegeneration, amyloid, and cognitive trajectory. *Neurology*, *95*(19), e2622–e2634. 10.1212/wnl.0000000000010531. 32732300 10.1212/wnl.0000000000010531 PMC7713731

[CR9] He, M., Li, Y., Zhou, L., Li, Y., Lei, T., Yan, W., & Chen, L. (2022). Relationships between Memory impairments and hippocampal structure in patients with subcortical ischemic vascular disease. *Frontiers in Aging Neuroscience*, *14*, 823535. 10.3389/fnagi.2022.823535. 35517055 10.3389/fnagi.2022.823535 PMC9062133

[CR10] Hou, G., Yang, X., & Yuan, T. F. (2013). Hippocampal asymmetry: Differences in structures and functions. *Neurochemical Research*, *38*(3), 453–460. 10.1007/s11064-012-0954-3. 23283696 10.1007/s11064-012-0954-3

[CR11] Huang, Y., Huang, L., Wang, Y., Liu, Y., Lo, C. Y. Z., & Guo, Q. (2022). Differential associations of visual memory with hippocampal subfields in subjective cognitive decline and amnestic mild cognitive impairment. *BMC Geriatrics*, *22*(1), 1–10. 35209845 10.1186/s12877-022-02853-7 PMC8876393

[CR12] Iadecola, C., Duering, M., Hachinski, V., Joutel, A., Pendlebury, S. T., Schneider, J. A., & Dichgans, M. (2019). Vascular cognitive impairment and dementia: JACC scientific expert panel. *Journal of the american college of cardiology*. *73*(25), 3326–3344. 10.1016/j.jacc.2019.04.034 PMC6719789 31248555

[CR13] Iglesias, J. E., Augustinack, J. C., Nguyen, K., Player, C. M., Player, A., Wright, M., & Wald, L. L. (2015). A computational atlas of the hippocampal formation using ex vivo, ultra-high resolution MRI: Application to adaptive segmentation of in vivo MRI. *J N*, *115*, 117–137. 10.1016/j.neuroimage.2015.04.042 PMC4461537 25936807

[CR14] Jack, C. R. Jr., Wiste, H. J., Weigand, S. D., Knopman, D. S., Mielke, M. M., Vemuri, P., & Petersen, R. C. (2015). Different definitions of neurodegeneration produce similar amyloid/neurodegeneration biomarker group findings. (1460–2156 (Electronic)). 10.1093/brain/awv283 PMC4655341 26428666

[CR15] Jia, J., Wei, C., Liang, J., Zhou, A., Zuo, X., Song, H., & Huang, L. (2016). The effects of DL-3-n-butylphthalide in patients with vascular cognitive impairment without dementia caused by subcortical ischemic small vessel disease: A multicentre, randomized, double-blind, placebo-controlled trial. *Alzheimer’s & Dementia*, *12*(2), 89–99. 10.1016/j.jalz.2015.04.010. 10.1016/j.jalz.2015.04.010 26086183

[CR16] Kang, D. W., Lim, H. K., Joo, S., Lee, N. R., & Lee, C. U. (2018). J. N. d., & treatment. The association between hippocampal subfield volumes and education in cognitively normal older adults and amnestic mild cognitive impairment patients. 143–152. 10.2147/NDT.S151659 PMC5757976 29379287

[CR17] Kesner, R. P. (2013). An analysis of the dentate gyrus function. *Behavioural Brain Research*, *254*, 1–7. 10.1016/j.bbr.2013.01.012. 23348108 10.1016/j.bbr.2013.01.012

[CR18] Kirino, T., & Sano, K. (1984). Selective vulnerability in the gerbil hippocampus following transient ischemia. *ACTA NEUROPATHOLOGICA*, *62*, 201–208. 6695554 10.1007/BF00691853

[CR19] Kril, J., Patel, S., Harding, A., & Halliday, G. (2002). Patients with vascular dementia due to microvascular pathology have significant hippocampal neuronal loss. *Journal of Neurology Neurosurgery & Psychiatry*, *72*(6), 747–751. 12023418 10.1136/jnnp.72.6.747 PMC1737900

[CR20] Li, X., Li, D., Li, Q., Li, Y., Li, K., Li, S., & Han, Y. (2016). Hippocampal subfield volumetry in patients with subcortical vascular mild cognitive impairment. *Scientific Reports*, *6*(1), 20873. 26876151 10.1038/srep20873 PMC4753487

[CR21] Liu, T., Deng, R., Wang, X., Liu, P., Xiao, Q. X., Liu, Q., & Zhang, Y. (2022). Mechanisms of hypoxia in the hippocampal CA3 region in postoperative cognitive dysfunction after cardiopulmonary bypass. *Journal of Cardiothoracic Surgery*, *17*(1), 106. 10.1186/s13019-022-01865-z. 35526011 10.1186/s13019-022-01865-z PMC9077938

[CR22] Long, X., Jiang, C., & Zhang, L. (2018). Morphological Biomarker Differentiating MCI Converters from Nonconverters: Longitudinal Evidence Based on Hemispheric Asymmetry. Behav Neurol, 2018, 3954101. 10.1155/2018/3954101. 10.1155/2018/3954101 PMC5884406 29755611

[CR23] Mueller, S. G., Schuff, N., Yaffe, K., Madison, C., Miller, B., & Weiner, M. W. (2010). Hippocampal atrophy patterns in mild cognitive impairment and Alzheimer’s disease. *Human brain mapping*, *31*(9), 1339–1347. 20839293 10.1002/hbm.20934 PMC2943433

[CR24] Nakashiba, T., Buhl, D. L., McHugh, T. J., & Tonegawa, S. (2009). Hippocampal CA3 output is crucial for ripple-associated reactivation and consolidation of memory. *Neuron*, *62*(6), 781–787. 10.1016/j.neuron.2009.05.013. 19555647 10.1016/j.neuron.2009.05.013 PMC2728553

[CR25] Nasreddine, Z. S., Phillips Na Fau - Bédirian, V., Bédirian, V., Fau - Charbonneau, S., Charbonneau, S., Fau - Whitehead, V., Whitehead, V. F., Collin, I., Collin, I. F., Cummings, J. L., & Chertkow, H. (2005). The Montreal Cognitive Assessment, MoCA: A brief screening tool for mild cognitive impairment. (0002-8614 (Print)). 10.1111/j.1532-5415.2005.53221.x 15817019

[CR26] Oschwald, J., Guye, S., Liem, F., Rast, P., Willis, S., Röcke, C., & Mérillat, S. (2019). Brain structure and cognitive ability in healthy aging: A review on longitudinal correlated change. *Reviews in the neurosciences*, *31*(1), 1–57. 31194693 10.1515/revneuro-2018-0096 PMC8572130

[CR27] Perrotin, A., de Flores, R., Lamberton, F., Poisnel, G., La Joie, R., de la Sayette, V., & Chételat, G. (2015). Hippocampal subfield volumetry and 3D surface mapping in subjective cognitive decline. (1875–8908 (Electronic)). 10.3233/JAD-150087 26402076

[CR28] Petersen, R. C., Ge Fau, S., Waring, S. C., Waring Sc Fau - Ivnik, R. J., Ivnik Rj Fau - Tangalos, E. G., Tangalos Eg Fau - Kokmen, E., & Kokmen, E. (1999). Mild cognitive impairment: clinical characterization and outcome. (0003-9942 (Print)). 10.1001/archneur.56.3.303 10190820

[CR29] Pluta, J., Yushkevich, P., Das, S., & Wolk, D. J. (2012). J. o. A. s. d. In vivo analysis of hippocampal subfield atrophy in mild cognitive impairment via semi-automatic segmentation of T2-weighted MRI. 31(1), 85–99. 10.3233/JAD-2012-111931 PMC3391337 22504319

[CR30] Puonti, O., Iglesias, J. E., & Van Leemput, K. J. N. (2016). Fast and sequence-adaptive whole-brain segmentation using parametric bayesian modeling. 143, 235–249. 10.1016/j.neuroimage.2016.09.011 PMC8117726 27612647

[CR31] Sarica, A., Vasta, R., Novellino, F., Vaccaro, M. G., Cerasa, A., & Quattrone, A. (2018). MRI asymmetry index of hippocampal Subfields increases through the Continuum from the mild cognitive impairment to the Alzheimer’s Disease. *Front Neurosci*, *12*, 576. 10.3389/fnins.2018.00576. 30186103 10.3389/fnins.2018.00576 PMC6111896

[CR32] Saygin, Z. M., Kliemann, D., Iglesias, J. E., van der Kouwe, A. J. W., Boyd, E., Reuter, M., & Augustinack, J. C. (2017). High-resolution magnetic resonance imaging reveals nuclei of the human amygdala: Manual segmentation to automatic atlas. *Neuroimage*, *155*, 370–382. 10.1016/j.neuroimage.2017.04.046. 28479476 10.1016/j.neuroimage.2017.04.046 PMC5557007

[CR33] Shi, F., Liu, B., Zhou, Y., Yu, C., & Jiang, T. (2009). Hippocampal volume and asymmetry in mild cognitive impairment and Alzheimer’s disease: Meta-analyses of MRI studies. *Hippocampus*, *19*(11), 1055–1064. 10.1002/hipo.20573. 19309039 10.1002/hipo.20573

[CR34] Sun, R., Ge, B., Wu, S., Li, H., & Lin, L. J. A. (2023). J. o. P. Optimal cut-off MoCA score for screening for mild cognitive impairment in elderly individuals in China: A systematic review and meta-analysis. 103691. 10.1016/j.ajp.2023.103691 37499366

[CR35] Suthana, N. A., Donix, M., Wozny, D. R., Bazih, A., Jones, M., Heidemann, R. M., & Bookheimer, S. Y. (2015). High-resolution 7T fMRI of human hippocampal subfields during associative learning. *Journal of Cognitive Neuroscience*, *27*(6), 1194–1206. 10.1162/jocn_a_00772. 25514656 10.1162/jocn_a_00772 PMC4417053

[CR36] Tatu, L., & Vuillier, F. (2014). Structure and vascularization of the human hippocampus. *Frontiers of Neurology and Neuroscience*, *34*, 18–25. 10.1159/000356440. 24777127 10.1159/000356440

[CR37] Thong, J. Y., Du, J., Ratnarajah, N., Dong, Y., Soon, H. W., Saini, M., & Qiu, A. (2014). Abnormalities of cortical thickness, subcortical shapes, and white matter integrity in subcortical vascular cognitive impairment. *Human Brain Mapping*, *35*(5), 2320–2332. 10.1002/hbm.22330. 23861356 10.1002/hbm.22330 PMC6869364

[CR38] Van Der Flier, W. M., Skoog, I., Schneider, J. A., Pantoni, L., Mok, V., Chen, C. L., & Scheltens, P. (2018). Vascular cognitive impairment. *Nature Reviews Disease Primers*, *4*(1), 1–16. 10.1038/nrdp.2018.3 29446769

[CR39] Vatrinet, R., Leone, G., De Luise, M., Girolimetti, G., Vidone, M., Gasparre, G., & Porcelli, A. M. (2017). The α-ketoglutarate dehydrogenase complex in cancer metabolic plasticity. *Cancer Metab*, *5*, 3. 10.1186/s40170-017-0165-0. 28184304 10.1186/s40170-017-0165-0 PMC5289018

[CR40] Woolard, A. A., & Heckers, S. (2012). Anatomical and functional correlates of human hippocampal volume asymmetry. *Psychiatry Research*, *201*(1), 48–53. 10.1016/j.pscychresns.2011.07.016. 22285719 10.1016/j.pscychresns.2011.07.016 PMC3289761

[CR41] Xu, J., Li, W., Bai, T., Li, J., Zhang, J., Hu, Q., & Wang, K. (2022). *Volume of hippocampus-amygdala transition area predicts outcomes of electroconvulsive therapy in major depressive disorder: High accuracy validated in two independent cohorts* (pp. 1–10). PSychological Medicine. 10.1017/S0033291722001337 35604047

[CR42] Yamamoto, Y., Hase, Y., Ihara, M., Khundakar, A., Roeber, S., Duering, M., & Kalaria, R. N. (2021). Neuronal densities and vascular pathology in the hippocampal formation in CADASIL. *Neurobiology of Aging*, *97*, 33–40. 10.1016/j.neurobiolaging.2020.09.016. 33130454 10.1016/j.neurobiolaging.2020.09.016 PMC7758782

[CR43] Yushkevich, P. A., Avants, B. B., Das, S. R., Pluta, J., Altinay, M., Craige, C., & Neuroimage, A. (2010). s. D. N. I. J. Bias in estimation of hippocampal atrophy using deformation-based morphometry arises from asymmetric global normalization: an illustration in ADNI 3 T MRI data. 50(2), 434–445. 10.1016/j.neuroimage.2009.12.007 PMC2823935 20005963

[CR44] Zotin, M. C. Z., Sveikata, L., Viswanathan, A., & Yilmaz, P. (2021). Cerebral small vessel disease and vascular cognitive impairment: From diagnosis to management. *Current opinion in neurology*. 34(2), 246. 10.1097/WCO.0000000000000913 PMC7984766 33630769

